# Histopathological investigation of glioblastomas resected under bevacizumab treatment

**DOI:** 10.18632/oncotarget.9387

**Published:** 2016-05-17

**Authors:** Ryota Tamura, Toshihide Tanaka, Keisuke Miyake, Yusuke Tabei, Kentaro Ohara, Oltea Sampetrean, Maya Kono, Katsuhiro Mizutani, Yohei Yamamoto, Yuichi Murayama, Takashi Tamiya, Kazunari Yoshida, Hikaru Sasaki

**Affiliations:** ^1^ Department of Neurosurgery, Keio University School of Medicine, Shinanomachi, Shinjuku-ku, Tokyo, Japan; ^2^ Department of Neurosurgery, Jikei University Kashiwa Hospital, Kashiwashita, Kashiwa-shi, Chiba, Japan; ^3^ Department of Neurosurgery, Kagawa University Hospital, Ikedo, Mituki-cho, Kita-gun, Kagawa, Japan; ^4^ Department of Neurosurgery, Japan Red Cross Medical Center, Hiroo, Shibuya-ku, Tokyo, Japan; ^5^ Division of Diagnostic Pathology, Keio University School of Medicine, Shinanomachi, Shinjuku-ku, Tokyo, Japan; ^6^ Division of Gene Regulation, Institute for Advanced Medical Research, Keio University School of Medicine, Shinanomachi, Shinjuku-ku, Tokyo, Japan; ^7^ Department of Neurosurgery, Jikei University Hospital, Nishishinbashi, Minato-ku, Tokyo, Japan

**Keywords:** bevacizumab, neoadjuvant, vascular normalization, hypoxia, glioma stem cell

## Abstract

To date, no clinical observations have been reported for histopathological changes in human gliomas under antiangiogenic treatment.

We collected six glioblastomas resected under bevacizumab treatment. Histopathological investigation was performed by hematoxilyn-eosin staining and immunohistochemistry for CD34, VEGF, VEGFR1/2, HIF-1α, CA9, and nestin as compared to eleven control glioblastomas to assess the differences in histological features, microvessel density, expression of VEGF and its receptors, tumor oxygenation, and status of glioma stem-like cells.

In the six tumors resected under bevacizumab, microvascular proliferation was absent, and microvessel density had significantly decreased compared with that of the controls. The expressions of VEGF and its receptors were downregulated in two cases of partial response. HIF-1α or CA9 expression was decreased in five of the six tumors, whereas the decreased expression of these markers was noted in only one of the 11 control glioblastomas. The expression of nestin significantly decreased in the six tumors compared with that of the controls, with the remaining nestin-positive cells being relatively concentrated around vessels.

We provide the first clinicopathological evidence that antiangiogenic therapy induces the apparent normalization of vascular structure, decrease of microvessel density, and improvement of tumor oxygenation in glioblastomas. These *in situ* observations will help to optimize therapy.

## INTRODUCTION

Bevacizumab (Bev) is a recombinant, humanized monoclonal antibody that acts against vascular endothelial growth factor (VEGF)-A and is being increasingly used for the treatment of recurrent high-grade gliomas. Data from randomized clinical trials suggest that Bev administration is associated with favorable event-free survival and improvement in the patient performance status,[1, 2] therefore, Bev has been approved for the newly diagnosed high-grade gliomas as well as for the recurrent tumors in Japan. Although the standard treatment for high-grade gliomas in Japan remains the so called Stupp regimen[3] because of lack of improvement in overall survival, possibility of Bev-related toxicity, etc., the upfront use of Bev combined with temozolomide and radiotherapy is among the therapeutic options for some cases such as in elderly patients and patients with unresectable tumors.

The proposed antitumor mechanisms of the antiangiogenic therapies include not only the inhibition of tumor angiogenesis but also indirect effects such as the depletion of vascular niches for cancer stem cells and modulation of immune responses [4, 5]. Moreover,antiangiogenic therapies have shown to normalize tumor vasculature, and thereby, increase tumor oxygenation with a possible improvement of radiosensitivity in experimental animals.[6, 7] However, to the best of our knowledge, there are no reports of changes in actual human gliomas under the control of antiangiogenic treatment. The *in situ* observation in surgical specimens of the effect of these therapies is likely crucial to understand the mechanism of action, predict treatment response, and clarify the molecular mechanism of resistance.

High-grade gliomas are hypervascular tumors with a frequent existence of intratumoral arterio**-**venous shunt, and such hypervascularity could occasionally hamper the safe resection of the tumors. On the basis of the high response rate of high-grade gliomas to Bev,[8, 9] the neoadjuvant use of Bev may be possibly useful for removal of those tumors on some occasions. In the current study, we included six cases in which Bev was used in the neoadjuvant setting either for a more safe surgical resection or as a consequence of the clinical course. In all the six cases, tumors were resected under the control of Bev before progression. To the best of our knowledge, this is the first report of histopathological evaluation of the effects of antiangiogenic therapies in patient-derived glioma specimens.

## RESULTS

### Case illustration and intraoperative findings

#### Case 1

**Figure 1:**
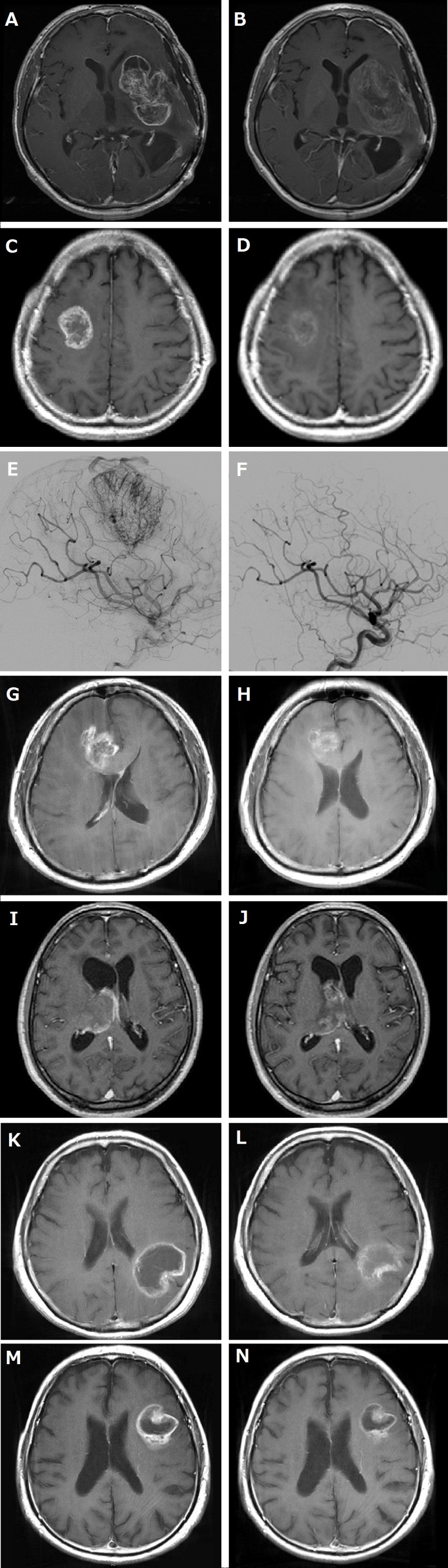
MRI and angiography of before and after neoadjuvant bevacizumab (Bev) A**-**D, G**-**N,: T1-weighted images with contrast enhancement, E and F: cerebral angiography. A, C, E, G, I, K and M: before Bev treatment. B, D, F, H, J, L and N: after Bev treatment (before removal). **A.** and **B.** Showing decrease of contrast enhancement and disappearance of peritumoral edema by three courses of neoadjuvant Bev in case 1. Change in sum of the products of the greatest perpendicular diameter (SPD) was −8%. **C.** and **D.** Showing volume decrease and improvement of contrast enhancement of the right premotor tumor in case 2 by two courses of neoadjuvant Bev and one course of temozolomide. SPD change was −52%. **E.** and **F.** Note that the prominent tumor staining in case 2 was disappeared by two courses of neoadjuvant Bev and one course of temozolomide. **G.** and **H.** Showing volume decrease and marked improvement of contrast enhancement as well as peritumoral edema of the right frontal-corpus callosum tumor in case 3 by one course of neoadjuvant Bev. SPD change was −61%. **I.** and **J.** Showing mild tumor volume decrease and improvement of contrast enhancement as well as hydrocepharus in case 4 by one course of neoadjuvant Bev. SPD change was −23%. **K.** and **L.** Showing volume decrease and marked improvement of contrast enhancement as well as peritumoral edema of the left parietal lobe tumor in case 5 by one course of neoadjuvant Bev. SPD change was −14%. **M.** and **N.** Showing volume decrease and marked improvement of contrast enhancement as well as peritumoral edema of the left frontal lobe tumor in case 6 by one course of neoadjuvant Bev. SPD change was −43%.

**Figure 2:**
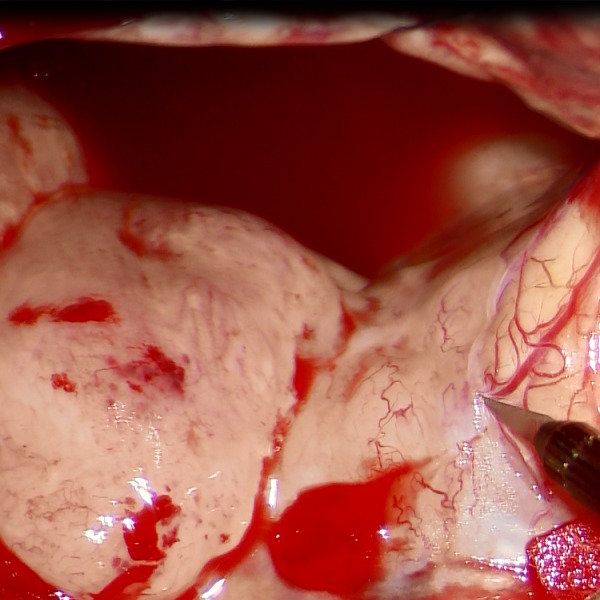
Intraoperative photograph during tumor removal in case 1 under neoadjuvant bevacizumab Note that the tumor is shown as milky-whitish mass, suggesting hypovascularity.

A 48-year-old Asian man presented with tumor regrowth (1st recurrence) after the initial chemoradiotherapy for left temporal anaplastic astrocytoma. A subtotal removal of the recurrent tumor was performed (2nd surgery), and the histopathological diagnosis was glioblastoma. Complete response was obtained by postoperative temozolomide; however, tumor recurrence was noted 8 months following surgery (2nd recurrence), and the patient was then treated with Bev. After the 1st course of Bev (10 mg/kg), although the tumor bulk remained stable, contrast enhancement almost disappeared, and the surrounding high-intensity area on T2/fluid-attenuated inversion recovery images reflecting peritumoral edema improved (RANO: SD, change in sum of the products of the perpendicular diameter (SPD) estimated by T1 weighted images with contrast enhancement: −8%) (Figure 1A and 1B).[10] Because of persistent headache and patient's hope for mass reduction, tumor removal after neoadjuvant Bev was planned and performed on day 36 of the 3rd course of Bev (continued effect of Bev was confirmed on MRI a day before operation, 3rd surgery). Intraoperatively, the tumor was milky-whitish; a quite different appearance as compared with the grayish to brownish previous tumor (Figure 2). Distinctiveness of the tumor margin was similar to that of the previous surgery with margins being mostly clear, although not clear at some deep parts. The tumor appeared hypovascular, and there was no particular difficulty in hemostasis. Because of lateral striate arteries penetrating the tumor, partial removal was performed (about 70%). BCNU wafer was placed on the resection margins. Suture removal was uneventfully done on day 30 after operation.

#### Case 2

A 49-year-old Asian man presented with an extremely hypervascular tumor in the right premotor area that was suspicious of glioblastoma (Figure 1C and 1E). Therefore, to decrease blood loss during surgery and to facilitate safe resection, tumor removal was planned after neoadjuvant Bev therapy. After two courses of Bev (10 mg/kg, day 0, 15) and one course of temozolomide (150 mg/m^2^, days 1**-**5), tumor volume was decreased, and angiography showed a disappearance of tumor stain (RANO: PR, SPD change: −52%) (Figure 1D and 1F). On day 21 of the 2nd course of Bev, gross total tumor removal was performed. Intraoperatively, the tumor was mostly yellowish, suggestive of necrosis. Tumor margins were mostly distinguished from the surrounding parenchyma. The tumor appeared hypovascular, and there was no difficulty in hemostasis. Suture removal was uneventfully done on day 12 after operation.

#### Case 3

A 55-year-old Asian man presented with a ring-enhanced lesion mainly located in the right frontal lobe with severe perifocal edema, and glioblastoma was suspected. To possibly decrease the extent of tumor and to facilitate operative radicality, tumor removal was planned after neoadjuvant Bev therapy. MRI after one course of Bev (10 mg/kg) revealed the marked improvement of the abnormal enhancement of the tumor as well as perifocal edema (RANO: PR, SPD change: **-**61%) (Figure 1G and 1H). On day 21 of the 1st course of Bev, gross total tumor removal was performed. Intraoperatively, the tumor presented as yellowish. Although the tumor was hypovascular, tumor margins were not very clear at most parts. There was no difficulty in hemostasis. Protoporphyrin IX fluorescence after the administration of 5-aminolevlinic acid was identified. Although there was a subcutaneous retention of the cerebrospinal fluid postoperatively, it gradually recovered. Suture removal was uneventfully done on day 14 after the operation.

#### Case 4

A 68-year-old Asian man presented with headache and disorientation. MRI showed irregularly enhanced lesion in the right thalamus with severe perifocal edema and hydrocephalus, and glioblastoma was suspected. Although the patient was initially conscious, he progressed rapidly to a state of drowsiness. Therefore, there was an urgent need to decrease tumor volume; hence, Bev was administered. After one course of Bev (10 mg/kg), MRI showed an improvement of perifocal edema and hydrocepharus as well as decrease of abnormal enhancement of the tumor (RANO: SD, SPD change: −23%) followed by improvement in consciousness (Figure 1I and 1J). On day 15 of the 1st course of Bev, endoscopic biopsy was performed. There was no difficulty in intraoperative hemostasis. Suture removal was uneventfully done on day 7 after operation.

#### Case 5

A right-handed, 77-year-old Asian female presented with a ring-enhanced lesion mainly located in the left parietal lobe, and glioblastoma was suspected. To possibly decrease the extent of the tumor located in the eloquent area and to minimize the post-operative complication, tumor removal was planned after neoadjuvant Bev therapy. MRI after one course of Bev (10 mg/kg) revealed the improvement of the abnormal enhancement of the tumor as well as perifocal edema (RANO: SD, SPD change: **-**14%) (Figure 1K and 1L). On day 26 of the 1st course of Bev, gross total tumor removal was performed. Intraoperatively, the tumor presented as brownish. Although the tumor was hypovascular, tumor margins were not very clear at most parts. There was no difficulty in hemostasis. Protoporphyrin IX fluorescence after the administration of 5-aminolevlinic acid was identified. Suture removal was uneventfully done on day 14 after the operation.

#### Case 6

A right-handed, 83-year-old Asian man presented with a ring-enhanced lesion in the left frontal lobe, and glioblastoma was suspected. To possibly decrease the extent of the tumor located in the language area and to minimize the post-operative complication, tumor removal was planned after neoadjuvant Bev therapy. MRI after one course of Bev (10 mg/kg) revealed the improvement of the abnormal enhancement of the tumor as well as perifocal edema (RANO: SD, SPD change: **-**43%) (Figure 1M and 1N). On day 27 of the 1st course of Bev, gross total tumor removal was performed. Intraoperatively, the tumor presented as dull red. Although the tumor was hypovascular, tumor margins were not very clear at most parts. There was no difficulty in hemostasis. Protoporphyrin IX fluorescence after the administration of 5-aminolevlinic acid was identified. Suture removal was uneventfully done on day 14 after the operation.

### Histological characteristics and vascular changes

#### Case 1 (Figure 3A to 3D)

Second surgery (before Bev treatment; Control 1): The tumor was mainly composed of medium sized astrocytic cells with increased cellularity and abundant mitotic figures. Numerous pseudopalisading necrosis was found and microvascular proliferation was predominantly present around the necrosis. Histopathological diagnosis was glioblatoma. The microvessel density was 32/5HPF (Table 1). The MIB-1 proliferative index was 58.3%, and the mitotic count was 18/10 HPF (Table 1).

Third surgery (after Bev treatment): The tumor excised under the control of Bev also showed astrocytic cell proliferation with nuclear anaplasia, and the form of tumor cells was essentially similar to that of the previous surgery. Pseudopalisading necrosis was occasionally observed. Vascular morphology was different from the previous specimen, and microvascular proliferation almost disappeared. Histopathological diagnosis was still glioblastoma. Microvessel density clearly decreased as compared with that of the tumor before Bev treatment (12.8 /5HPF) (Table 1). The mitotic count did not change (17/10HPF). MIB-1 index was 20% (Table 1).

**Table 1:** Results of immunohistochemical analyses of the 6 tumors resected under control of neoadjuvant Bev as compared to 11 control glioblastomas

*Cases (response in SPD change)*	*Microvessel density by CD34 staining (mm*^2^*)*	*Mitotic count (/10HPF)*	*MIB-1 index(%)*	*VEGF-A*	*VEGFR1*	*VEGFR2*	*HIF-1α*	*CA9*	*Nestin %*
*Case 1 (−8%)*	12.8	17	20	++	++	+	++	+	16.6
*Case 2 (−52%)*	14.2	5	3	-	-	+-	+	+-	16.2
*Case 3 (−61%)*	19.4	12	10.9	-	-	+-	-	-	6.7
*Case 4 (−23%)*	27.6	7	6	++	++	+	-	-	18
*Case 5 (−14%)*	15.4	8	20	++	++	+	-	+	29.1
*Case 6 (−43%)*	17.6	3	70	+	+	+	-	+	39.4
*Control 1 (before BEV in Case 1)*	32	18	58.3	++	+	+	++	++	34.4
*Control 2*	21	10	67.3	++	+	+	+	+	40.1
*Control 3*	47	7	36.2	++	+	+	++	++	95.1
*Control 4*	68	26	54.7	++	+	+	+	++	97.4
*Control 5*	91.2	7	6.9	++	+	+	++	++	82.4
*Control 6*	91.6	13	34	++	+	+	++	++	88.6
*Control 7*	50.2	8	23.3	++	++	+	++	++	96
*Control 8*	52.8	9	23.8	++	++	+	++	++	91.3
*Control 9*	55.6	9	24.9	++	+	+	++	+	68.9
*Control 10*	30.6	8	41.3	++	++	+	+	+-	83.3
*Control 11*	44.4	5	5	++	++	+	++	++	92.5

The microvessel density and nestin positive cell ratio are significantly decreased in the 6 tumors after neoadjuvant Bev compared to control tumors (p=0.002, p=0.000028, respectively, *t* test).

SPD: sum of the product of the perpendicular diameters, BEV: bevacizumab, HIF-1α:hypoxia inducible factor-1α, VEGF: vascular endothelial growth factor, VEGFR: vascular endothelial growth factor receptor. VEGF-A was assessed as the following; ++: diffuse intense staining, +: diffuse faint staining, -: negative staining. VEGFR1 and VEGFR2 were assessed as the following: ++: staining in both vascular endothelial cells and tumor cells, +: staining only in vascular endothelial cells, -: negative staining. HIF-1α was assessed as the following: ++: diffuse cytoplasmic or nuclear staining in tumor cells, +: staining only in a subset of tumor cells, -: negative staining .CA9 was assessed as the following: ++: universal strong expression around necrotic regions, +: occasional expression around necrotic regions, −: negative staining.

#### Case 2 (Figure 3E and 3F)

The great portion of the resected tissue was occupied by coagulative necrosis. The viable neoplastic cells of mild to moderate nuclear atypia were observed around the necrotic area with moderate cellularity, equivalent to grade II or III glioma. Neither pseudopalisading necrosis nor microvascular proliferation was observed. Mitotic count was 5/10 HPF and MIB-1 index was 3% (Table 1). Microvessel density was 14.2/5HPF.

#### Case 3 (Figure 3G and 3H)

The resected tumor showed a characteristic distribution of tumor cells, with a predominant accumulation around the vessels and hypocellular parenchyma (vascular co-option). The endothelial cells of the vessels were somewhat hypertrophied; however, microvascular proliferation or glomeruloid structure was not observed. Tumor cells showed nuclear pleomorphism with increased mitosis and occasional multinucleated cells. Pseudopalisading necrosis was absent. Histopathological diagnosis was high-grade glioma. Mitotic count was 12/10 HPF. Micovessel density was 19.4/5HPF (Table 1). MIB-1 index was 11%.

#### Case 4 (Figure 3I)

The tumor was composed of spindle or polygonal shaped cells with increased cellularity and nuclear pleomorphisms. The vessels of this tumor were regularly shaped, and the vascular walls were thin. Neither microvascular proliferation or pseudopalisading necrosis were observed. Histopathological diagnosis was high-grade glioma. Mitotic activity was not very high (7/10 HPF), and MIB-1 index was 6% (Table 1). Microvessel density was 27/5HPF (Table 1).

#### Case 5 (Figure 3J and 3K)

The resected tumor showed a characteristic distribution of tumor cells, with a predominant accumulation around the vessels and hypocellular parenchyma (vascular co-option) as with Case 3. Microvascular proliferation was not observed. Tumor cells showed nuclear pleomorphism with increased mitosis. Pseudopalisading necrosis was absent. Histopathological diagnosis was high-grade glioma. Mitotic count was 8/10 HPF. Micovessel density was 15.4/5HPF (Table 1). MIB-1 index was 20%.

#### Case 6

The resected tumor showed a characteristic distribution of small to medium sized tumor cells, with a predominant accumulation around the vessels and hypocellular parenchyma (vascular co-option) as with Case 3 and 5. Tumor cells showed nuclear pleomorphism. Microvascular proliferation was not observed. Pseudopalisading necrosis was absent. Histopathological diagnosis was high-grade glioma. Mitotic count was 3/10 HPF. Micovessel density was 17.6/5HPF (Table 1). MIB-1 index was 70%.

#### Control glioblastomas

MRI of the eleven control, newly diagnosed glioblastomas are shown in Supplementary Figure 1 (Figure S1). Mean microvessel density of the control glioblastomas was 50.2/5HPF, and the density of the six tumors resected under neoadjuvant Bev (mean 16.5/5HPF) was significantly decreased compared with the control tumors (Table 1, *p* = 0.002). The same was also true when a comparison was made between the control tumors and the five tumors, excluding the biopsy case (case 4) in which the tumor sample may not represent the whole picture of the tumor (*p* = 0.003). In contrast, there was no significant difference in the mitotic count between the six tumors after Bev treatment and control tumors (mean, after Bev treatment: 7.5/10 HPF, control: 9/10 HPF, *p* = 0.457). There was no significant difference in the MIB-1 proliferative indices between the six tumors resected under neoadjuvant Bev and control tumors (mean: after Bev treatment (15.5%) *vs*. control (34%) *p* = 0.276) (Table 1).

**Figure 3:**
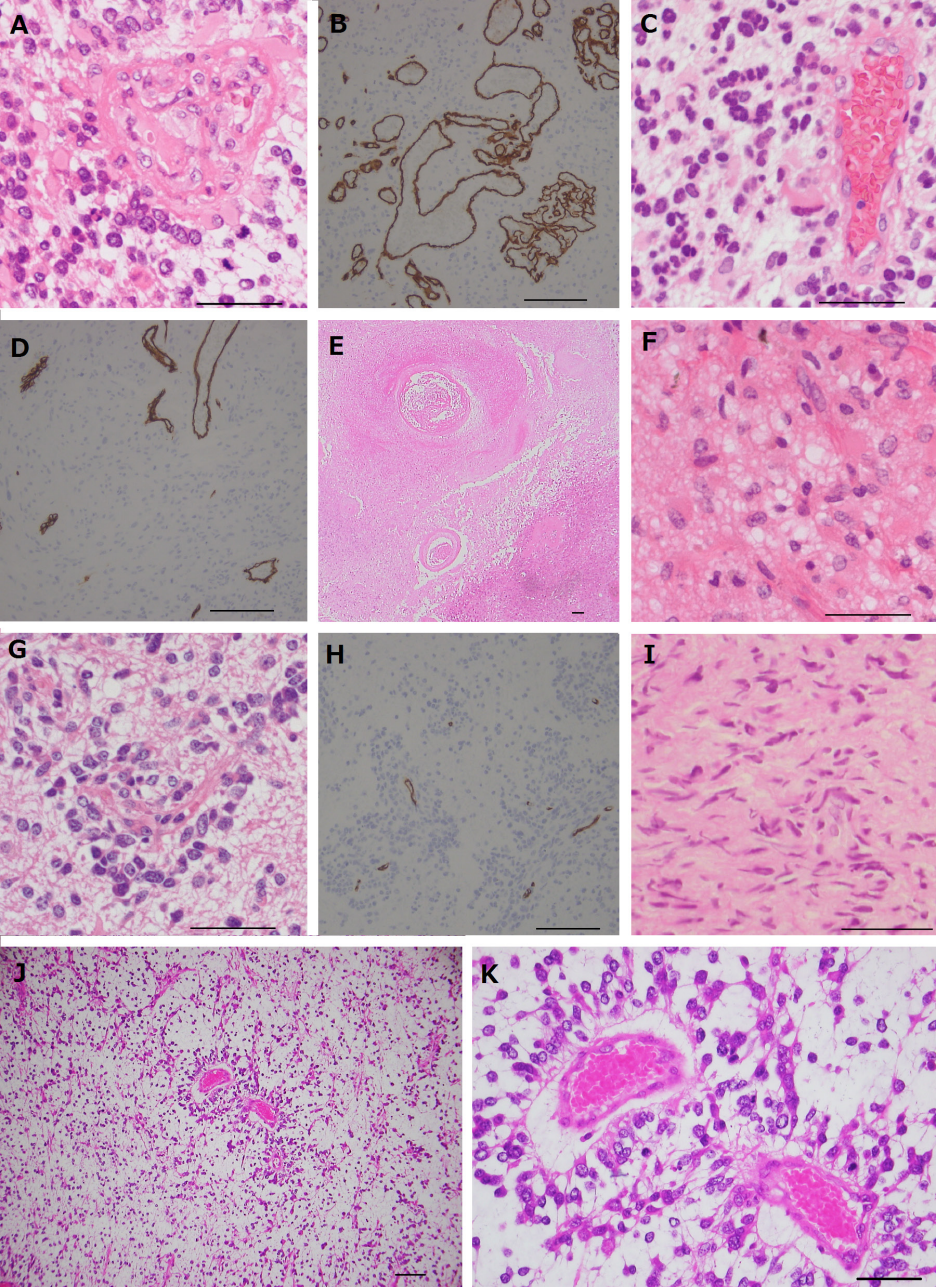
Photomicrographs of the tumors resected under neoadjuvant bevacizumab (Bev) A, C, E, F, G, I, J, and K: hematoxylin**-**eosin staining, B, D, and H: immunohistochemical analysis of CD34. **A.** and **B.** Before Bev treatment in case 1, show hypercellular tumor with medium sized astrocytic cells and microvascular proliferation. Microvessel density as assessed by CD34 immunohistochemical analysis is 32/5HPF. (A: original magnification ×400, The magnification bar: 50 μm, B: original magnification ×200, The magnification bar: 100 μm). **C.** and **D.** After Bev treatment in case 1. Note that endothelial cell hyperplasia is improved, and microvascular proliferation has disappeared. Microvessel density is clearly decreased (12.8/5HPF) (C: original magnification ×400, The magnification bar: 50 μm, D: original magnification ×200, The magnification bar: 100 μm). **E.** and **F.** After Bev treatment in case 2. In low power field (E), the majority of the tumor is occupied by coagulative necrosis. (original magnification ×40, The magnification bar: 100 μm). The neoplastic cells show mild to moderate degree of nuclear atypia equivalent to grade II or III glioma, and microvessels are apparently normal without microvascular proliferation (F). (original magnification ×400, The magnification bar: 50 μm). **G.** and **H.** After Bev treatment in case 3. Note that the tumor cells are predominantly accumulated around vessels (vascular co-option). The microvascular proliferation is not observed. Microvessel density is 19.4/5HPF (G: original magnification ×400, The magnification bar: 50 μm, H: original magnification ×200, The magnification bar: 100 μm). **I.** After Bev treatment in case 4 showing spindle or polygonal shaped cells with increased cellularity and nuclear pleomorphism. Vessels of this tumor are regularly shaped, and the vascular walls are thin. (original magnification ×400, The magnification bar: 50 μm). **J.** and **K.** After Bev treatment in case 5. Note that the tumor cells are predominantly accumulated around vessels (vascular co-option). The microvascular proliferation is not observed. (J: original magnification ×100, The magnification bar: 100 μm, K: original magnification ×400, The magnification bar: 50 μm).

### Immunohistochemical analyses of VEGF and VEGFRs

#### VEGF-A (Figure 4A to 4D)

The positivity of VEGF-A staining in tumor cytoplasm or stroma was assessed as the following: ++, diffuse intense staining; +, diffuse faint staining; −, negative staining.

Among the six tumors resected under neoadjuvant Bev, staining was diffuse intense (++) in cases 1, 4 and 5, and diffuse faint in case 6, whereas staining was negative (−) in cases 2 and 3 (Table 1). In contrast, the expression of VEGF-A was diffusely intense (++) in all control tumors, including the tumor before Bev treatment in case 1 (Table 1).

#### VEGFR1, 2 (Figure 4E to 4H)

The staining positivity of VEGFR1 and VEGFR2 on endothelial or tumor cell membrane/cytoplasm was assessed as the following: ++, staining in both vascular endothelial cells and tumor cells; +, staining only in vascular endothelial cells; −, negative staining.

Among the six tumors resected under neoadjuvant Bev, the expression of VEGFR 1 was not observed either in vascular endothelial cells or tumor cells in cases 2 and 3 (staining: −). In contrast, VEGFR1 expression was observed in the endothelial cells and a subset of tumor cells in cases 1, 4 and 5 (staining: ++), and only in vascular endothelial cells in case 6. The expression of VEGFR 2 was observed only in vascular endothelial cells in all the six cases; however, it appeared to be weak to negative in cases 2 and 3 (cases 1, 4, 5 and 6: +; cases 2 and 3: +−).

VEGFR1 was expressed in the endothelial cells and a subset of tumor cells in four (staining: ++), and only in vascular endothelial cells in seven of the 11 control cases (staining: +). VEGFR2 was expressed in vascular endothelial cell in all the control cases (staining: +).

**Figure 4:**
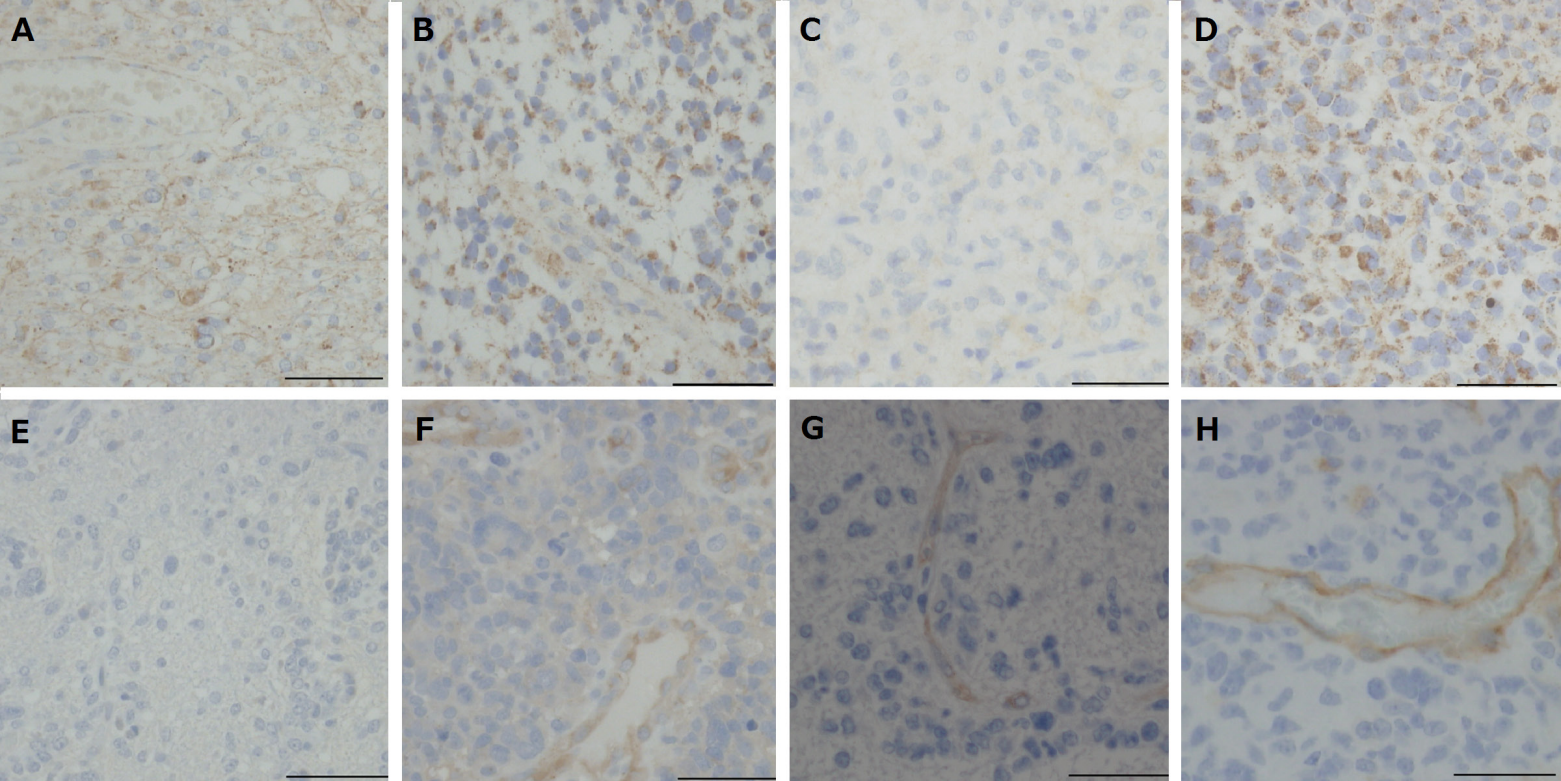
Immunohistochemical analysis for VEGF and VEGFRs in tumors resected under neoadjuvant bevacizumab (Bev) as compared with that in control glioblastomas **A.** Before Bev treatment in case 1, shows diffuse staining for VEGF. (VEGF-A, original magnification ×400. The magnification bar: 50 μm). **B.** After Bev treatment in case 1, shows diffuse staining for VEGF (VEGF-A, original magnification ×400. The magnification bar: 50 μm). **C.** After Bev treatment in case 2. The tumor is negative for VEGF. (VEGF-A, original magnification ×400. The magnification bar: 50 μm). **D.** Control glioblastoma, showing diffuse staining for VEGF. (VEGF-A, original magnification ×400. The magnification bar: 50 μm). **E.** After Bev treatment in case 3. The expression of VEGFR1 is not observed either in vascular endothelial cells or tumor cells. (VEGFR1, original magnification ×400. The magnification bar: 50 μm). **F.** Control glioblastoma, showing staining for VEGFR1 in vascular endothelial cells. (VEGFR1, original magnification ×400. The magnification bar: 50 μm.). **G.** After Bev treatment in case 3, shows faint staining for VEGFR2 in vascular endothelial cells. (VEGFR2, original magnification ×400. The magnification bar: 50 μm). **H.** Control glioblastoma, showing strong staining for VEGFR2 in vascular endothelial cells. (VEGFR2, original magnification ×400. The magnification bar: 50 μm).

### Immunohistochemical analyses of tumor oxygenation (Figure 5)

The expression of HIF-1α was predominantly detected in the nuclei of tumor cells around necrosis and was also found in the tumor cells not directly adjacent to necrotic area. Degree of expression was assessed as the following: ++, expression in > 10% of tumor cells; +, expression in ≤10% of tumor cells; −, negative staining.

Among the six tumors resected under neoadjuvant Bev, the expression of HIF-1α was not observed in cases 3, 4, 5 and 6 (staining: −) and was observed occasionally among tumor cells adjacent to the large coagulative necrosis in case 2 (staining: +). In case 1, the expression of HIF-1α was detected in many of the perinecrotic tumor cells (staining: ++). Among the control tumors, the expression of HIF-1α was observed occasionally in three of the 11 tumors (staining: +), and in many tumor cells in the other eight (staining: ++).

The membranous expression of CA9 was predominantly found in the perinecrotic tumor cells as previously reported.[11] The degree of the expression was assessed as the following: ++, universal strong expression around necrotic regions; +, occasional expression (typically around necrotic regions); −, negative staining.

The expression pattern of CA9 was consistent with that of HIF-1α in the majority of the cases. Among the six tumors resected under neoadjuvant Bev, the expression of CA9 was negative or nearly negative in cases 2, 3, and 4 (case 2: +−; cases 3 and 4: −). The expression of CA9 was observed in the tumor cells around a few, not all of the necrotic regions in case 1 (staining: +), and was found in a few spots in cases 5 and 6 despite absence of pseudopalisading necrosis (staining: +). In the 11 control glioblastomas, the expression of CA9 was universally observed around many necrotic regions in eight (staining: ++), occasionally observed in two, and was nearly negative in one.

**Figure 5:**
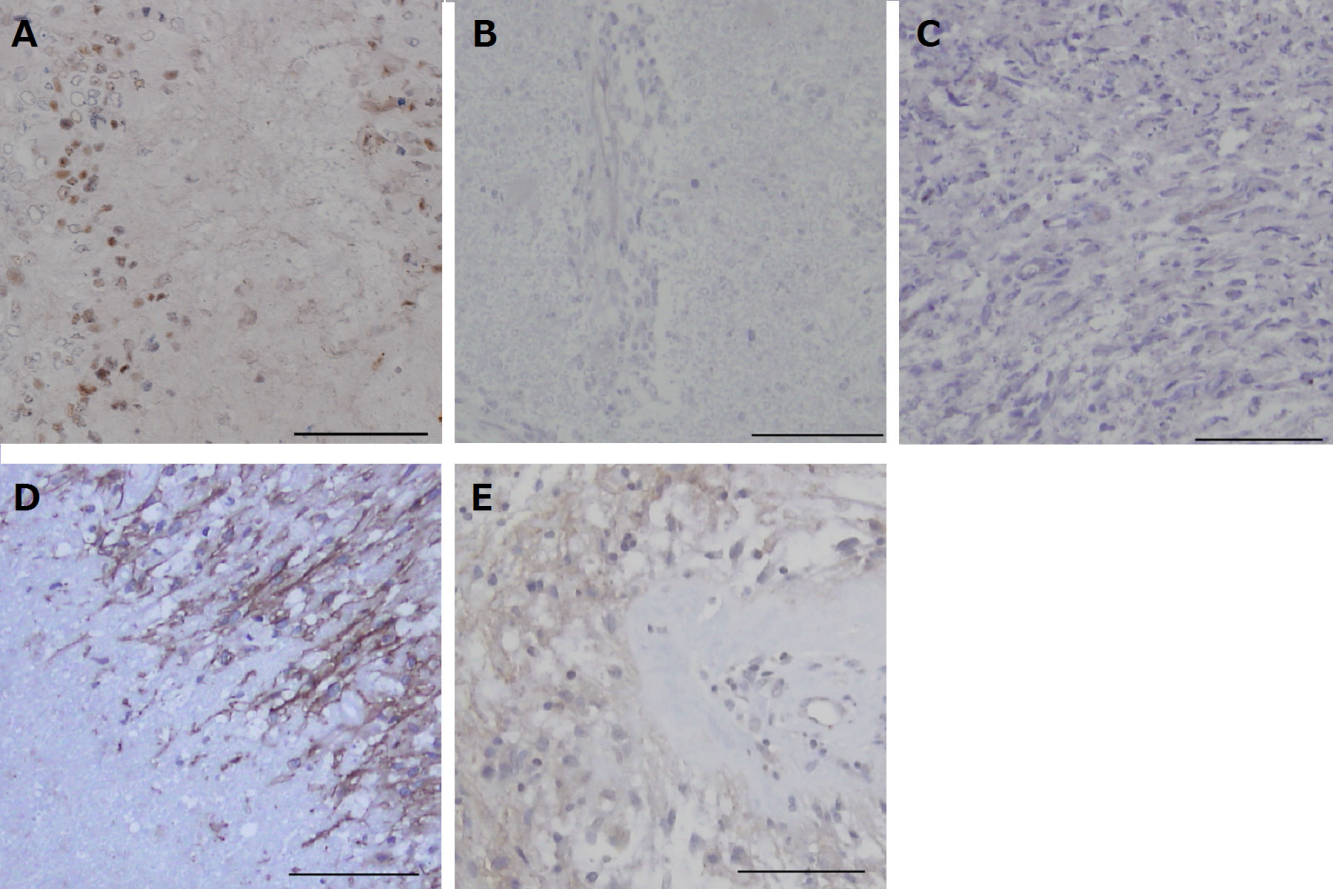
Immunohistochemical analysis for HIF-1α and CA9 in the tumors resected under neoadjuvant bevacizumab (Bev) as compared with that in control glioblastomas **A.** After Bev treatment in case 1, HIF-1α is expressed in many tumor cells predominantly around necrotic regions. (HIF-1α, original magnification ×200. The magnification bar: 100μm). **B.** After Bev treatment in case 3. The expression of HIF-1α is not observed. (HIF-1α, original magnification ×200. The magnification bar: 100 μm). **C.** After Bev treatment in case 4. The expression of HIF-1α is not observed. (HIF-1α, original magnification ×200. The magnification bar: 100 μm). **D.** Control glioblastoma showing strong membranous staining of CA9 in the perinecrotic tumor cells. (CA9, original magnification ×200. The magnification bar: 100 μm). **E.** After Bev treatment in case 2, CA9 is only weakly expressed in a few areas adjacent to large necrosis. (CA9, original magnification ×200. The magnification bar: 100 μm).

### Nestin immunohistochemistry (Figure 6)

Nestin-positive cells were significantly less frequent in the tumors resected under neoadjuvant Bev than the control glioblastomas. (positive cell ratio, mean 17.3% *vs*. 88.6%, *p* = 0.000028). This was also true when a comparison was made between the control tumors and the five tumors, excluding the biopsy case (case 4) (*p* = 0.000106). However, there were still numerous nestin-positive tumor cells in the six tumors, and these positive cells were relatively concentrated around vessels.

**Figure 6:**
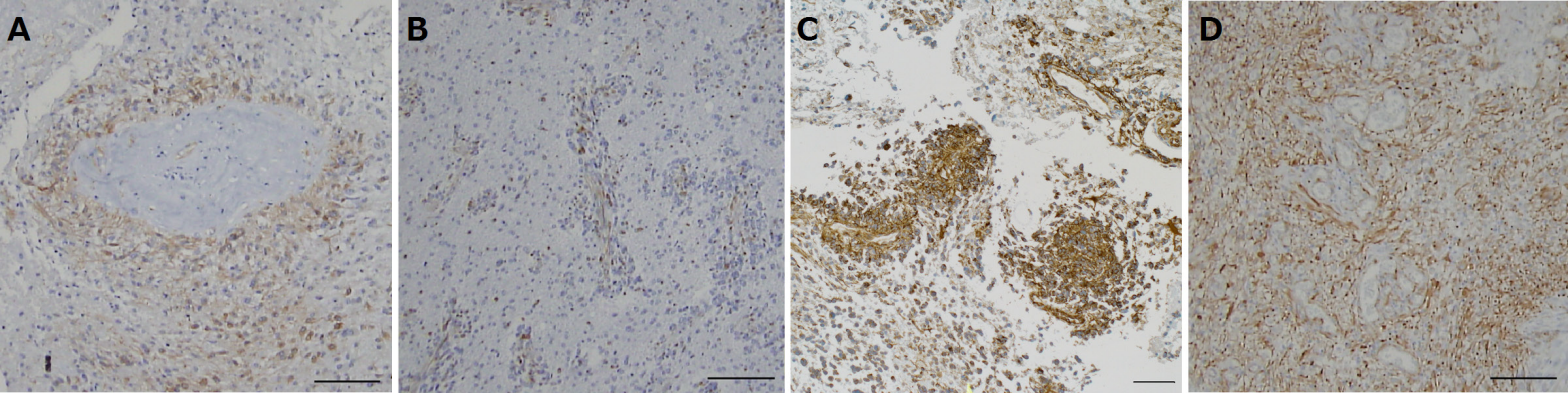
Immunohistochemical analysis for nestin in tumors resected under neoadjuvant bevacizumab (Bev) as compared with that in control glioblastomas **A.** After Bev treatment in case 2. The expression of nestin is predominantly found in the tumor cells around a vessel (the vessel wall shows fibrous thickening and the vascular lumen is lined by nestin-positive endothelial cells). Nestin-positive cell ratio is 16.2%. (nestin, original magnification ×100. The magnification bar: 100 μm). **B.** After Bev treatment in case 3. Note that the majority of the nestin-positive cells are found around vessels. Nestin-positive cell ratio is 6.7%. (nestin, original magnification ×100. The magnification bar: 100 μm). **C.** After Bev treatment in case 6. Note that the expression of nestin is predominantly found around vessels. Nestin-positive cell ratio is 39.4%. (nestin, original magnification ×100. The magnification bar: 100 μm). **D.** Control glioblastoma showing diffuse strong staining in tumor cells and vascular endothelial cells. Nestin-positive cell ratio is 73%. (nestin, original magnification ×100. The magnification bar: 100 μm).

## DISCUSSION

All of the six tumors resected under neoadjuvant Bev demonstrated characteristic histological changes such as disappearance of microvascular proliferation and significant reduction of microvessel density, suggesting the normalization of the vascular structure. Importantly, these observations closely resemble those reported in mice orthotopic models [6, 7, 12] and were found regardless of treatment response. Although there are some similar studies in the literature that dealt with tumor tissue before and after Bev, [13, 14] the changes in vascular morphology were inconsistent or not mentioned. The tumors examined in those previous studies were recurrent that progressed after Bev treatment; therefore the post-Bev histological findings have been likely affected by changes associated with tumor progression. Thus, to the best of our knowledge, the present study provides the first *in situ* confirmation of the normalization of vascular structure on human glioma specimens under the control of Bev. One may argue that the paired comparison between pre and post Bev tissue was performed in only one of the 6 tumors. However, the pre-Bev MR images and angiography of the six tumors were obviously typical for glioblastoma, and the unusual, striking histological characteristics in the post-Bev six tumors were most likely to be caused by Bev treatment. Moreover, tumor response to temozolomide is usually slow, and maximal response is typically noted after six cycles even in the 1p19q-codeleted gliomas.[15] Therefore, the histological changes in case 2, the case treated with temozolomide and Bev, was also considered mainly because of Bev treatment.

We showed that the expression of VEGF-A was negative in cases 2 and 3, and was decreased in case 6, whereas its expression was equivalent in cases 1, 4, and 5 to that of the control glioblastomas. Similarly, we did not observe the expression of VEGFR1 in cases 2 and 3, whereas VEGFR1 appeared somewhat upregulated in cases 1, 4, and 5 compared to control glioblastomas. Also, expression of VEGFR2 appeared weak in cases 2 and 3 as compared to that of cases 1, 4, 5, 6 and controls. Although further studies are warranted, these observations are intriguing because of correlations to radiological response of each tumor to neoadjuvant Bev treatment. Bev was very effective in cases 2 and 3, moderately effective in case 6, and less effective in cases 4 and 5, whereas the tumor size remained unchanged in case 1. One may expect that VEGF is universally downregulated in tumors under the control of Bev. However, in less favorable responders (cases 1, 4 and 5), we found VEGF and its receptors to be equally expressed to control tumors despite Bev dosing being the same in all six cases (10 mg/kg). In contrast, in good responders (cases 2 and 3), not only VEGF but also its receptors are downregulated. Although the reason for this phenomenon is unclear, we could suggest the followings as possible mechanism. First, negative feedback mechanisms are present in poorly responsive tumors leading to the augmented expression of VEGF-A from tumor cells. Second, affinity of Bev varies depending on VEGF-A genotype. Indeed, some VEGF polymorphisms are reported to be associated with improved overall survival in clinical trials with regimens including Bev.[16] However, it is currently unknown whether the apparent correlation of the expression of VEGF and its receptors with therapeutic response is causal or a consequence. Further studies exploring these relationships are warranted.

Angiogenesis inhibitors are shown to increase tumor oxygenation by the normalization of tumor vasculature in mice orthotopic models or by MRI analysis in patients.[6, 17, 18] In the present study, the expressions of HIF-1α and CA9, endogenous markers for tumor hypoxia, were not detected or were very low in four and three of the six cases for each (cases 3, 4, 5, and 6 for HIF-1α and cases 2, 3, 4 for CA9), and, therefore, the improvement of tumor oxygenation was suggested in five of the 6 tumors resected under Bev treatment. Indeed, pseudopalisading necrosis was not observed in these five tumors, with the exception of case 1 in which tumor response was minimal. On the other hand, the improved oxygenation was observed in only one (control 10) of the 11 control glioblastomas. A previous study demonstrated *via* MRI analysis that cediranib (a pan-VEGF receptor tyrosine kinase inhibitor) induces the improvement of perfusion and oxygenation in only a subset of glioblastoma patients despite the decrease of contrast enhancement and brain edema in nearly all patients.[17] Our data is in line with this previous observation. In the present study, we observed improvements in tumor oxygenation in the five better responders, despite the normalization of vascular structure as well as decreases of microvessel density being clearly demonstrated in all six cases. Although the reason for this discrepancy is currently unknown, it is possible that in the less favorable responders, the intratumoral pressure may remain high because of the still abundant tumor cells; therefore, tumor hypoxic condition may not be improved. The possible correlation of the improvement of oxygenation and response to Bev treatment warrants further investigation.

Cancer stem cells (CSCs) are considered to be the major source of tumor recurrence after radiation and chemotherapy, and the literature suggests that targeting CSCs is one of the promising approaches to overcome treatment resistance of cancers.[19–21] Additionally, stem cell niches maintaining CSCs are also considered to be important targets to treat glioblastoma,[22] and currently, two types of stem cell niches are reported: perivascular niche and hypoxic niche.[4, 23] In the present study, the expression of nestin, a marker of glioma stem cell-like phenotype, was found to be decreased in the six tumors resected under neoadjuvant Bev treatment as compared with control glioblastomas. However, numerous nestin-positive tumor cells remained after Bev treatment, with relative concentration around vessels (Figure 6A, 6B, 6C). These observation as well as vascular co-option of the tumor cells noted in cases 3, 5, and 6 may suggest that improvement of a low -oxygen environment by Bev treatment could make it difficult to maintain a hypoxic niche. In contrast, the perivascular niche is still likely present around the apparently “normalized” vasculature under antiangiogenic therapy, and the other therapeutic approach or combination therapy may be necessary to eradicate perivascular CSCs.

In conclusion, histopathological investigation of six glioblastomas resected under neoadjuvant Bev treatment demonstrated the apparent normalization of vascular structure, reduction of microvessel density, and the improvement of tumor oxygenation. The expression of VEGF-A and VEGFRs appears to negatively correlate with radiologic responses. The number of glioma stem-like cells decreased after neoadjuvant Bev, however, there were still numerous nestin-positive cells predominantly around vessels. These observations will help to better understand the mechanism of action of antiangiogenic therapy, to elucidate the mechanism of resistance to Bev, and to allow the optimization of therapy for high-grade gliomas. Further studies are warranted.

## MATERIALS AND METHODS

Histopathological evaluation was performed on six gliomas resected under the control of Bev, and the findings were correlated with therapeutic response. Each of these 6 cases was treated in four collaborative institutes from January 2014 to December 2015 in Japan (Keio University Hospital, Kagawa University Hospital, Jikei University Kashiwa Hospital, and Japan Red Cross Medical Center). The six tumors were examined for histological features, vascular structure/microvessel density, VEGF/VEGF receptors (VEGFRs), tumor hypoxic condition, and glioma stem-like cells, and the results were compared with those of the eleven control glioblastomas [ten consecutive newly diagnosed glioblastomas resected in 2014 (all primary glioblastoma) and one glioblastoma before Bev treatment in case 1 (recurrence of anaplastic astrocytoma)]. This translational research has been approved by the Institutional Review Board at each of the four institutes. Written informed consent was obtained from all the sixteen patients, and the following analyses were performed at Keio University School of Medicine.

Histopathological analyses were performed on 4-μm sections of formalin-fixed, paraffin-embedded tissue of the 17 tumors from 16 patients. All of the available tissue blocks were examined for each of the following analyses. Histological characteristics were assessed using hematoxylin and eosin staining. Tumor proliferative activity was evaluated using immunohistochemistry with anti-Ki67 antibody (1:200, MIB-1, Dako, Glostrup, Denmark) as previously described.[24] For assessment of microvessel density, the tissue sections were screened using CD34 immunohistochemistry (1:100, anti-CD34 antibody, QBEnd 10, DAKO) at low power field (x40), and 5 most vascularized areas (hot spots) were selected. Counting of microvessels was performed on these areas at high power field (HPF: x200, 0.95mm^2^).[6, 14, 25]

The expression of VEGF-A, VEGF receptor (VEGFR) 1 (flt-1), and VEGFR 2 (KDR/flk-1) was examined using immunohistochemistry with anti-VEGF-A antibody (1:200, JH121, MERICK MILLIPORE, Tokyo, Japan), anti-VEGFR1 antibody (1:100, AF321, R&D SYSTEMS, MN, USA), and anti-VEGFR2 antibody (1:600, 55B11, Cell Signaling Technology, Tokyo, Japan).[14, 25, 26]

Hypoxia-inducible factor-1α (HIF-1α), a subunit of HIF-1 transcriptional factor, is regulated depending on tissue oxygen concentration and is negligibly detected under normoxic condition. Carbonic anhydrase 9 (CA9) is a transmembrane glycoprotein that catalyzes the reversible hydration of carbonic dioxide to carbonic acid. The expression of CA9 is tightly regulated by HIF-1; therefore, CA9 is also considered an endogenous hypoxia marker in tumors.[11, 26, 27] Tumor hypoxic condition was evaluated using immunohistochemistry with anti-HIF1α antibody (1:100, H-206, Santa Cruz Biotechnology, Texas, USA) and anti-CA9 antibody (1:50, H-120, Santa Cruz Biotechnology).

Nestin was first described as a neural stem/progenitor cell marker and is also considered a marker of glioma cells with a stem cell-like phenotype.[28, 29] To examine the effect of Bev on glioma stem-like cells, expression of nestin was examined using immunohistochemistry with an anti-nestin antibody (1:100, 10C2, Chemicon, Tokyo, Japan).

In immunohistochemical analyses, antigen retrieval was performed in citrate buffer (pH 6 for VEGFR1, nestin, and CA9), or in Tris buffer (pH 9 for CD34, VEGF-A, VEGFR2, and HIF-1α) using microwave irradiation or autocrave (HIF-1α), and the products were visualized with peroxidase-diaminobenzidine reaction. The expression of VEGF-A, VEGFR1/2, HIF-1α, and CA9 was assessed by consensus of four authors (RT, TT, KO, and HS). The expression of nestin was assessed as a positive cell ratio analyzed in more than 1000 tumor cells from more than three areas, showing the representative appearance of each tumor.

Student's t-test was used to compare microvessel density, mitotic count, and staining indices of MIB-1 and nestin between the six tumors resected under the control of Bev and the eleven control glioblastomas. Analyses were performed with IBM SPSS statistics.[19]

## SUPPLEMENTARY MATERIALS FIGURES


