# Supplementary material for: Histopathological investigation of glioblastomas resected under bevacizumab treatment
Source: Oncotarget. 2016 May 17;7(32):52423–35. doi: 10.18632/oncotarget.9387 (PMC5239563; doi:10.18632/oncotarget.9387)
Supplement: Supplementary file 1 [file oncotarget-07-52423-s001.pdf]

Histopathological investigation of glioblastomas resected under bevacizumab treatment

Supplementary Material

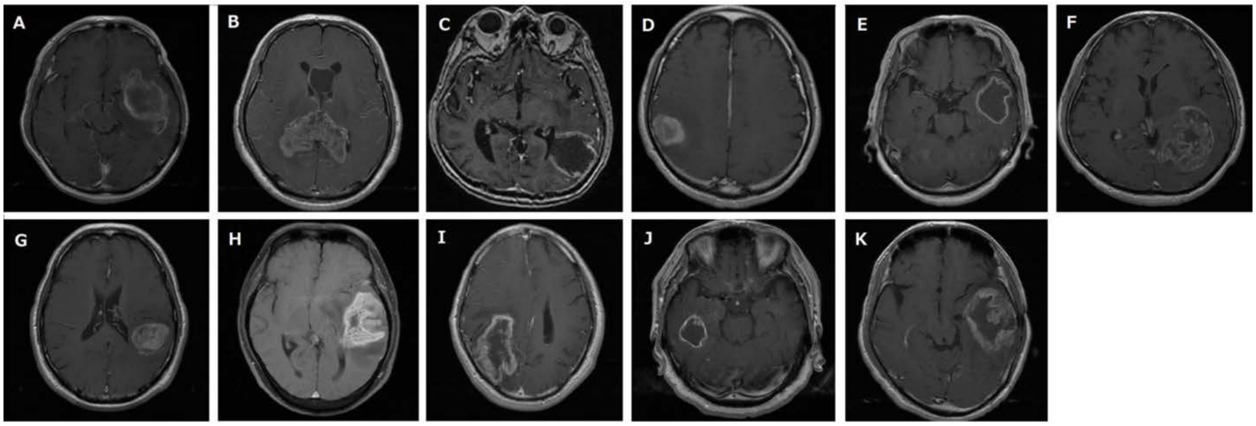

Supplementary Figure S1: T1-weighted MRI with contrast enhancement of 11 control cases. A (control 1): 48-year-old Asian man, B (control 2): 51-year-old Asian man, C (control 3): 77-year-old Asian man, D (control 4): 44-year-old Asian man, E (control 5): 72-year-old Asian woman, F (control 6): 57-year-old Asian man, G (control 7): 64-year-old man, H (control 8): 55-year-old Asian man, I (control 9): 66-year-old Asian man, J (control 10): 79-year-old Asian man, K (control 11): 59-year-old Asian man.
